# Unconscious processes in psychotherapy and supervision: Difficulties in the supervisory relationship

**DOI:** 10.1192/j.eurpsy.2025.2151

**Published:** 2025-08-26

**Authors:** L. Mustoo Başer

**Affiliations:** Psychology, International University of Sarajevo, 71210, Bosnia and Herzegovina

## Abstract

**Introduction:**

Supervision is recognized as having a vital role in the professional development of psychotherapists. The way it is implemented has a direct impact on the quality of the educator’s learning process, as well as on the quality of psychotherapy provided to clients. Supervision is a complex and dynamic practice, requiring supervisors to conceptualize the thoughts, feelings and behavior of their educators and the educator’s clients, as well as selective attention to the complexly layered interpersonal dynamics between clients, educators and themselves.

**Objectives:**

Emphasizing the possibility of the appearance of difficulties and their aspects and increasing awareness of these concepts can greatly help supervisors and educators in the formation of ways and approaches that can facilitate better communication and the formation of a more professional relationship as significantly influencing the main result of the quality of psychotherapy and the process of professional development.

**Methods:**

During the training of mental health professionals, it is crucial to ensure that the supervision process is carried out according to a legal, ethical and competent approach, such as informed consent and a supervision contract, and it is necessary to take into account elements such as supervisor and educator competencies, awareness of the challenges of diversity and multiculturalism, personal and professional boundaries, multiple contexts of relationship between supervisor and educator, evaluation and feedback. Through the supervisor’s dilemmas, ambiguities in the perceptions of supervision educators, success is reflected through the analysis of personal contribution, of unconscious interpersonal and developmental dynamics, and access to one’s own limits.

**Results:**

The importance of clinical supervision is evidently recognized by almost all licensing boards and accrediting bodies for the mental health professions, requiring that educators receive psychotherapeutic, clinical supervision as part of their training, and ongoing supervision is often necessary to maintain professional licensure. An integrative approach of incorporating innovative research results, as well as consolidating them with already established factors, is key in leading psychotherapeutic training, research, supervision, as well as the client’s treatment itself towards increased effectiveness. A significant contribution to the literature would be enabled by future research dealing with issues of difficulties in supervision through the prism of educators.

**Conclusions:**

This paper focuses on understanding the significance of the supervision process thorugh the theoretical perspective, approaching the concept of difficulties by the prism of supervisors and educators, and the practical implications of successful supervision.

**Disclosure of Interest:**

None Declared

